# Vertical Beta-Diversity of Bacterial Communities Depending on Water Stratification

**DOI:** 10.3389/fmicb.2020.00449

**Published:** 2020-03-19

**Authors:** Wan-Hsuan Cheng, Hsiao-Pei Lu, Chung-Chi Chen, Sen Jan, Chih-hao Hsieh

**Affiliations:** ^1^Taiwan International Graduate Program–Earth System Science Program, Academia Sinica, Taipei, Taiwan; ^2^Taiwan International Graduate Program–Earth System Science Program, National Central University, Taoyuan, Taiwan; ^3^Institute of Oceanography, National Taiwan University, Taipei, Taiwan; ^4^Department of Biotechnology and Bioindustry Sciences, National Cheng Kung University, Tainan, Taiwan; ^5^Department of Life Science, National Taiwan Normal University, Taipei, Taiwan; ^6^Institute of Ecology and Evolutionary Biology, Department of Life Science, National Taiwan University, Taipei, Taiwan; ^7^Research Center for Environmental Changes, Academia Sinica, Taipei, Taiwan; ^8^National Center for Theoretical Sciences, Taipei, Taiwan

**Keywords:** vertical beta diversity, 16S rRNA gene, water mixing, stratification, environmental gradients

## Abstract

Many studies indicate that variation of marine bacterial beta diversity in the horizontal dimension is mainly attributable to environmental and spatial effects. However, whether and how these two effects drive bacterial beta diversity in the vertical dimension remains unclear, especially when considering seasonal variation in the strength of water stratification. Here, we used 78 paired bacterioplankton community samples from surface and deep chlorophyll maximum (DCM) layers along a transect in the Kuroshio region east of Taiwan across multiple seasons. Variance partitioning was used to evaluate the mechanisms driving the vertical beta diversity between surface-DCM bacterioplankton communities during weak stratification periods (i.e., spring and fall) versus strong stratification periods (i.e., summer). During strong periods of stratification, vertical beta diversity was shaped by both environmental and spatial effects; notably, the strength of stratification played an important role in enhancing environmental dissimilarity and creating a barrier to dispersal. In contrast, during periods of weak stratification, environmental effects dominate, with a non-significant spatial effect due to mixing. Variation of vertical beta diversity for bacterioplankton communities in the Kuroshio region east of Taiwan was structured by different mechanisms across seasons, and was further dependent on stratification strength of the water column.

## Introduction

Bacteria are ubiquitous in the ocean (Flombaum et al., 2013; Sunagawa et al., 2015) and play a are critical to ecosystem functions such as biogeochemical cycling of elements (Falkowski et al., 1998). Therefore, efforts to investigate how bacterial species composition varies among sites (so-called beta diversity) have been increasing (Hanson et al., 2012; Sunagawa et al., 2015; Milici et al., 2016; Wu et al., 2018). Generally, beta diversity can be explained by two effects (Soininen, 2012). The first is the environmental effect: bacterial communities are strongly shaped by local environmental variables such as nutrient concentration and temperature (Cram et al., 2015; Sunagawa et al., 2015; Lawes et al., 2016); thus, greater physicochemical dissimilarity results in higher beta diversity (Webb et al., 2002; Yu et al., 2018). The second is the spatial effect: bacterial communities exhibit dispersal limitation resulting from geographic isolation (Hanson et al., 2012); hence, geographic isolation such as geographic distance can lead to higher beta diversity (Lindstrom and Langenheder, 2012).

The majority of bacterial beta diversity studies have focused on the horizontal dimension (Martiny et al., 2006; Hanson et al., 2012; Soininen, 2012; Sunagawa et al., 2015; Milici et al., 2016; Wu et al., 2018). However, the three-dimensional of the ocean, has prompted more recent examination of beta diversity in the vertical dimension (hereafter, vertical beta diversity) (Treusch et al., 2009; Qian et al., 2011; Giovannoni and Vergin, 2012; Chow et al., 2013; Li et al., 2018). In addition to physicochemical dissimilarity and geographic (vertical) distance, these recent studies suggest that that vertical beta diversity may also be influenced by physical attributes such as water column stratification (Treusch et al., 2009; Qian et al., 2011; Giovannoni and Vergin, 2012; Chow et al., 2013; Li et al., 2018). For example, at the Bermuda Atlantic Time-series Study (BATS) site, during summer seasons when the stratification is strong, the deep chlorophyll maximum (DCM) with high nutrient concentrations is formed, in contrast to the surface water with low nutrient concentrations. Consequently, bacteria in the euphotic zone were found to be vertically separated into an oligotrophic community in surface water and a eutrophic community in the DCM. During the other seasons when stratification is weaker, water in the euphotic zone becomes more vertically homogeneous and a unified bacterial community was found instead (Treusch et al., 2009; Giovannoni and Vergin, 2012). In contrast, at the Hawaii Ocean Time-series (HOT) site where water stratification is prolonged throughout the whole year, discrete surface and DCM communities were persisted the euphotic zone (Giovannoni and Vergin, 2012). Moreover, a study in the South China Sea where stratification is typically strong indicated that beta diversity was more vertically pronounced relative to the horizontal dimension, suggesting that water column stratification did enhance beta diversity (Li et al., 2018). Therefore, the strength of stratification should be considered as part of a clearer understanding of the mechanisms determining vertical beta diversity.

Stratification prevents mixing between water layers. Strong stratification can create dispersal barriers (Baltar and Arístegui, 2017), thus influencing vertical beta diversity through spatial effects with vertical distance. In addition, stratification can enhance physicochemical dissimilarity between water layers (Boucher et al., 2006) and thus affects vertical beta diversity through environmental effects. However, no study has attempted to investigate how vertical beta diversity is driven by different levels of spatial and environmental effects, which are both mediated by stratification strength. Moreover, as stratification strength can change through time, it is possible that the mechanisms driving vertical beta diversity varies temporally. Therefore, to understand which mechanisms shape vertical beta diversity, it is important to investigate the relative importance of environmental and spatial effects in the context of seasonal variation in water stratification strength.

**FIGURE 1:**
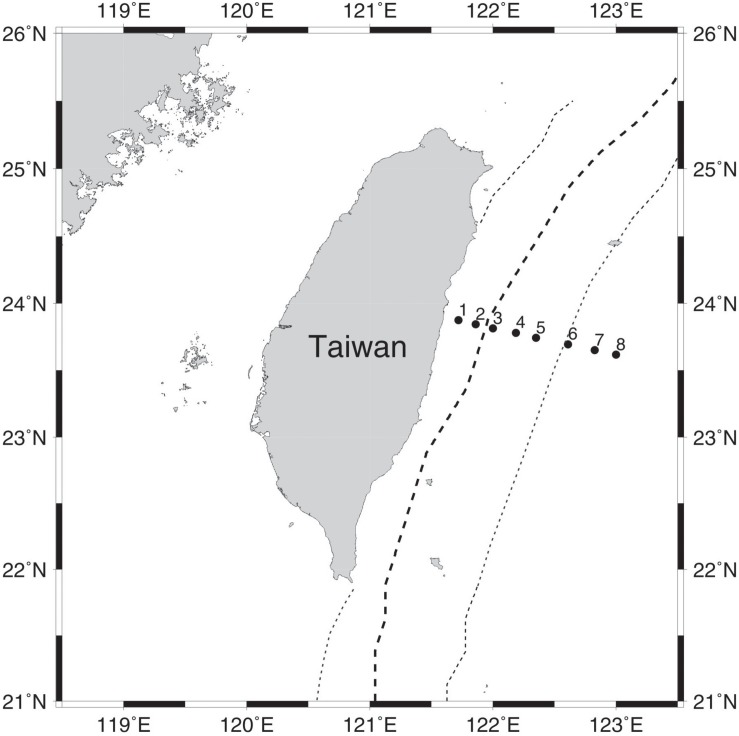
Map illustrating sampling stations along a transect perpendicular to the Kuroshio east of Taiwan. The bold dashed line indicates the mean position of the maximum current; the dotted line indicates the average width of the Kuroshio.

In this study, we focused on disentangling how vertical beta diversity of bacterioplankton communities were affected by environmental and spatial effects as mediated by seasonal variation in stratification strength. To achieve this goal, samples were collected during five surveys across multiple seasons along a repeated transect in the Kuroshio region east of Taiwan (Figure 1). This study transect is part of the ongoing Observations of the Kuroshio Transports and their Variability (OKTV) project (Jan et al., 2015), as a counterpart of the Origins of the Kuroshio and Mindanao Current (OKMC) project (Qiu et al., 2013; Lien et al., 2014). Following on previous studies (Giovannoni and Vergin, 2012; Chow et al., 2013), we sampled both the surface and DCM layers in order to examine the vertical dimension. To investigate the influence of seasonal variation of stratification strength, spring and fall were assigned as weakly stratified periods, whereas summer was assigned as strongly stratified periods (see section “Materials and Methods” for the detailed definition). We hypothesize that strong stratification enhances both environmental and spatial effects. That is, during the strongly stratified periods, both environmental and spatial effects significantly contributed to vertical structuring of bacterioplankton communities; while during the weakly stratified periods, the spatial and environmental effects would be largely weakened due to water mixing. To test this hypothesis, the environmental and spatial effects resulted from relative contribution of physicochemical dissimilarity, vertical distance, and stratification strength were quantified using three-way variation partitioning (Legendre et al., 2005) (see section “Materials and Methods”).

## Materials and Methods

### Sample Collection

A total of 78 samples were collected in June of 2013, March, July, and November of 2014, and March of 2015 by GoFlo bottles equipped with conductivity, temperature and depth profiler (CTD profiler, Sea-Bird Electronics, Bellevue, WA, United States) along a transect of eight stations in the Kuroshio region between 121.72° and 123° E at 23.75° N east of Taiwan (Figure 1). To compare bacterial communities in the vertical dimension, we collected water samples from both the surface and deep chlorophyll *a* maximum (DCM) layers. During each sampling event, ∼20 L water at each layer was first passed through a 1.2 μm-pore-size polycarbonate filter (Millipore, United States) to exclude eukaryotes and other larger particles, and then through a 0.2 μm-pore-size filter to retain the bacterioplankton community. The sample processing was completed within 4 h of water collection (Yeh et al., 2015). After filtration, the 0.2 μm-pore size filters were stored in liquid nitrogen onboard and then at −20°C until molecular analysis.

Temperature and salinity were recorded by a CTD profiler, and vertical profiles of density were calculated at each sampled station. Seawater was sampled at eight depths (2–5, 10, 30, 50, 100, 150, 200, and 250 m) with Go-Flo bottles. Dissolved organic nitrogen (DIN), phosphate (PO_4_), silicate (SiO_2_), and chlorophyll *a* concentrations were measured according to standard protocols (Gong et al., 2003).

### DNA Extraction, PCR Amplification and Sequencing

Total DNA was extracted from the 0.2 μm-pore size filter using the Meta-G-Nome^TM^ DNA Isolation Kit (Epicentre Biotechnologies, Madison, WI, United States) according to the manufacturer’s instructions. Extracted DNA was used as template of polymerase chain reaction (PCR) to amplify V5-V6 region (∼300 bp) of 16S rRNA gene using the forward primer FIA-787F (5′-[forward index adaptor]-ATTAGATACCCNGGTAG-3′) and reverse primer RIA-1046R (5′-[reverse index adaptor]-CGACAGCCATGCANCACCT-3′) (Cai et al., 2013). PCR was performed with two-steps to gain better reproducibility and consistent results (Berry et al., 2011). In detail, the first-step PCR conditions were an initial denaturation at 95°C for 3 min; 25 cycles of 94°C for 30 s, 55°C for 45 s, 72°C for 1 min; and a final extension at 72°C for 2 min. The second-step PCR conditions were at 95°C for 3 min, 8 cycles of 95°C for 30 s, 55°C for 30 s, 72°C for 30 s; and a final extension at 72°C for 5 min. The amplicons from PCR were sequenced through Illumina Miseq platform, producing 2 × 300 bp paired-end reads.

### Sequence Processing

To reduce sequencing errors, low-quality sequences (<Q30) were excluded using Trimmomatic 0.36 (Bolger et al., 2014). The paired-end reads were then assembled with the 100 bp overlapping region and processed through quality controls by PANDAseq. To minimize the effects of random sequencing errors, filtering parameters, including (i) non-overlapping paired-reads; (ii) sequences <120 nucleotides; (iii) with incomplete or incorrect primer sequences; and (iv) with more than one undetermined nucleotide, were used (Yang et al., 2018). Qualified sequences were processed by the Quantitative Insights Into Microbial Ecology (QIIME) pipeline (Caporaso et al., 2010). Operational taxonomic units (OTUs) were classified with 97% similarity using Sumaclust (Mercier et al., 2013). Taxonomy assignments was based on Silva 119 database (Quast et al., 2013). OTUs appearing only once were excluded to prevent PCR-induced error.

### Estimation of Vertical Beta Diversity

Vertical beta diversity was based on Bray-Curtis dissimilarity between paired surface and deep chlorophyll maximum (DCM) communities of a sampled station. To ensure consistency in sequencing effort among samples, the community was based on the average of 100 subsampled OTU tables with the sequence number rarefied to 5900 (considering that the minimum sequence number among the samples is 5906).

### Vertical Physicochemical Dissimilarity

Six environmental variables (temperature, DIN, PO_4_, SiO_2_, salinity and chlorophyll *a* concentration) were used to compute physicochemical dissimilarity. Each variable was normalized to zero mean and unit variance and before the Euclidean distance between the paired samples was calculated.

### Vertical Distance

The vertical distance was simply the difference in sampling depth between the surface (3–5 m) and DCM layers of each site. The sampling depth of the DCM layer was determined by inspection of the fluorescence profile during CTD descent.

### Strength of Stratification

To assess the strength of stratification, we calculated the buoyancy frequency (BF, or Brunt–Väisälä frequency) for each meter up to the depth of DCM layer:

$$B\,F=\sqrt{-\frac{g}{\rho_{0}}\,\frac{d\,\rho }{d\,z}}$$

where *g* (9.8 m s^–2^) is the local acceleration of gravity, ρ_0_ is a reference seawater density (1025 kg m^–3^ in this study) and *z* is depth in vertical axis. Since a larger BF indicates stronger stratification, we used the maximum value of BF as an indicator of the strength of water column stratification for each sampling event (Turner, 1973).

### Spatial-Temporal Variation of Bacterioplankton Communities

In order to explore how bacterioplankton communities were associated with seasonal, horizontal and vertical variation, we presented the similarity of bacterioplankton communities using de-trended correspondence analysis (DCA) based on the average of 100 subsampled OTU tables.

### Separation of Water Weakly Stratified Periods From Strongly Stratified Periods

We assigned cruises in spring and fall (March 2014, November 2014, and March 2015) as ‘weakly stratified’ periods and cruises in summer (June 2013, July 2014) as ‘strongly stratified’ periods. To justify our assignment of weakly and strongly stratified periods, the strength of stratification of these periods was compared using Wilcoxon signed-rank test. Besides, to further understand whether weakly and strongly stratified periods exhibit distinct environmental and biological features, vertical physicochemical dissimilarity and vertical beta diversity of the two periods were also compared using Wilcoxon signed-rank test respectively.

### Evaluation of Vertical Bacterioplankton Beta Diversity

To evaluate how vertical beta diversity was determined in weakly and strongly stratified periods, we carried out the analysis separately for each period (Supplementary Table S1). Specifically, linear regression was used to relate the vertical beta diversity with physicochemical dissimilarity, vertical distance and the strength of stratification. To determined how much of the variation in the bacterioplankton vertical beta diversity is explained by the physicochemical dissimilarity ([Env]), vertical distance ([Dist]) and stratification strength ([BF]), three-way variation partitioning (Legendre et al., 2005) was employed to decompose the total variation of beta diversity into pure physicochemical dissimilarity component ([Env|Dist + BF]), pure stratification component ([BF|Env + Dist]), and pure distance component ([Dist|Env + BF]). In particular, the environmental effect is represented by physicochemical dissimilarity ([Env]), while the spatial effect is represented by the pure stratification component ([BF|Env + Dist]) and/or pure distance component ([Dist|Env + BF]). The relative contribution of each component was calculated by the adjusted variance and statistically tested by permutation test, except for the intersection and residuals. The shared fractions may yield negative values as a statistical consequence of the adjustment related to the number of predictors. In such cases, the negative results should be interpreted as zero, indicating that the explanatory variables explain less variation than normal variables would (Legendre, 2008).

### Computation

All statistical analyses were conducted in R (version 3.3.5)^1^. Detrended corresponding analysis (DCA), estimation of distance matrix and variation partitioning were performed with the package ‘vegan’ (Jari Oksanen et al., 2018).

## Results

### Hydrography Varies Between Strongly and Weakly Stratified Periods

**FIGURE 2:**
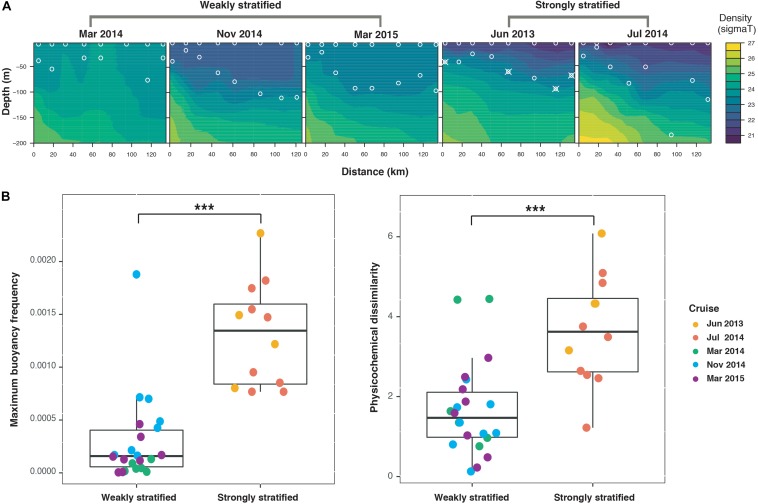
Hydrographical properties along the sampling transect at different sampling periods. **(A)** Contour plots of density (sigma T) within 200 m water depth. Circles indicate sampling locations (surface and deep chlorophyll maximum layers). Circles combined with crosses indicate the samples with contamination (see Supplementary Figure S2); therefore, these contaminated samplings and their corresponding surface samples were both excluded from the remainder of the analysis. **(B)** Boxplots illustrating the strength of stratification (quantified by maximum buoyancy frequency) and vertical environmental dissimilarity. The strong stratification periods exhibited significantly greater maximum buoyancy frequency and greater physicochemical dissimilarity than the weak stratification periods (Wilcoxon signed-rank test, ***P < 0.001).

In the Kuroshio region east of Taiwan, the hydrography exhibited distinct seasonal patterns: summer as strongly stratified periods, and spring and fall as weakly stratified periods (Figure 2). During the strongly stratified periods, water stratification (according to the maximal buoyancy frequency of water column, see section “Materials and Methods”) was due to the large temperature gradient caused by high sea surface temperature (Supplementary Figure S1). In contrast, during the weakly stratified periods, the strength of stratification was significantly weaker relative to that of summer (Wilcoxon signed-rank test, *P* < 0.001) (Figure 2B). Moreover, while the vertical dissimilarity in physicochemical variables was relatively small in the weakly stratified periods compared to the strongly stratified periods (Wilcoxon signed-rank test, *P* < 0.001), appreciable vertical physicochemical gradients existed during both periods (Figure 2B). Specifically, during the strongly stratified periods, the vertical dissimilarity in physicochemistry was correlated with the layer-specific differences in chlorophyll *a*, silicate and salinity. By contrast, during the weakly stratified periods, the vertical dissimilarity of physicochemical variables was related to the difference in chlorophyll *a*, silicate, and phosphate (Supplementary Figure S2).

### General Patterns of Bacterioplankton

After quality checking and filtering, a total of 903,383 sequences were obtained from 78 samples. At the phylum level, *Proteobacteria* (∼59%), *Actinobacteria* (∼13%), *Bacteroidetes* (∼7%), *Planctomycetes* (∼7%), *Deferribacteres* (∼5%), *Firmicutes* (∼5%), and *Cyanobacteria* (∼3%) accounted for about 98% of the total sequences. However, four samples (all from DCM layers in station 1, station 5, station 7, and station 8) in Jun 2013 contained abnormally high proportion of *Firmicutes (Bacilli)* (Supplementary Figure S3); these were considered as contaminations (Janssen, 2006; Wu et al., 2010) and these four sites (thus eight samples) were removed from further analyses. In addition, two stations (st 3, st 6 in Mar 2014) in which DCM coincided with the surface layer were also excluded. As a consequence, 68 out of the 78 samples (34 sites) with a total number of 7821 OTUs were retained for our analysis.

### Bacterioplankton Assemblages Among Sampling Sites and Seasons

**FIGURE 3:**
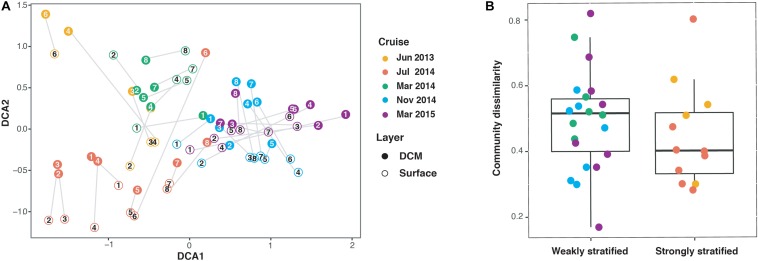
**(A)** Ordination biplot illustrating the association among samples based on detrended Correspondence Analysis (DCA) of the bacterioplankton communities for different cruises and depth layers. The numbers indicate sampling stations (c.f. Figure 1). Each gray line represents vertical beta diversity as measured using Bray-Curtis distance between the paired surface and deep chlorophyll maximum (DCM) communities of a sampling. **(B)** Boxplots indicating that no significant difference in vertical beta diversity existed between the weakly and strongly stratified periods (Wilcoxon signed-rank test, *P* = 0.23).

The remaining 68 bacterioplankton communities based on OTU level were clustered according to sampling cruises, which were further grouped into the strongly and weakly stratified periods (Figure 3A). Separation of communities among different depths (surface versus DCM) was also apparent for each sampling cruise. However, mean vertical beta diversity did not show a significant difference between the strongly and weakly stratified periods (Wilcoxon signed-rank test, *P* = 0.23) (Figure 3B).

### Relative Contribution of Environmental and Spatial Effects in Explaining Vertical Beta Diversity

**FIGURE 4:**
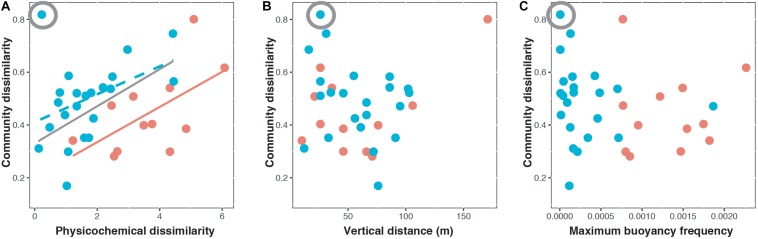
Bray–Curtis dissimilarity of paired surface and deep chlorophyll maximum communities in relation to **(A)** physicochemical dissimilarity, **(B)** vertical distance, and **(C)** maximum buoyancy frequency during the weakly stratified periods (blue) and strongly stratified periods (orange). The circled symbol indicates the potential outlier. The relationship without the potential outlier was presented by the gray line. The solid line represents a significant relationship (*P* < 0.05) while the dashed line represents a marginally significant relationship (*P* < 0.1).

During strongly stratified periods (Figure 4 and Table 1), both environmental and spatial effects were found to influence vertical beta diversity, supporting our hypothesis. The results of three-way variation partitioning indicated that the environmental effect ([Env]) appeared to be the major component contributing to the vertical beta diversity (*adjusted R*^2^ = 28%, *P* = 0.039, Table 1). However, the pure effect of physicochemical dissimilarity ([Env|Dist+BF]) was not significant (*adjusted R*^2^ = 2%, *P* = 0.29, Table 1). Instead, vertical beta diversity was fundamentally determined by the combination of stratification strength and vertical distance (multivariate linear regression, *adjusted R*^2^ = 45%, *P* = 0.029). These results indicated that the strength of stratification enhanced physicochemical dissimilarity, which in turn drove vertical beta diversity. In addition, we found that the pure effect of vertical distance ([Dist|Env+BF]) is marginally significant (*adjusted R*^2^ = 8.9%, *P* = 0.052) (Table 1), whereas the pure effect of stratification ([BF|Env+Dist]) is not significant (*adjusted R*^2^ = 25%, *P* = 0.15) (Table 1). These results suggested that vertical beta diversity can be influenced by dispersal limitation contributed by the pure effects of vertical distance ([Dist|Env+BF]) but to a much lesser extent by the pure effects of stratification strength ([BF|Env+Dist]). In summary, during strongly stratified periods, vertical beta diversity of bacterioplankton was determined by both environmental and spatial effects. The stratification strength mainly contributed to enhancing physicochemical gradients but also to resulted, some extent, to elevated dispersal limitation.

**TABLE 1:** Results of variation partitioning against the physicochemical dissimilarity [Env], vertical distance [Dist] and stratification strength [BF] components based on the Bray-Curtis distance.

	[Env]		[Dist]		[BF]		[Env|Dist+BF]		[Dist|Env+BF]		[BF|Env+Dist]	
	*Var.*	*P*	*Var.*	*P*	*Var.*	*P*	*Var.*	*P*	*Var.*	*P*	*Var.*	*P*
Strongly stratified	**0**.**28**	**0**.**039**	−0.099	0.12	0.15	0.92	0.016	0.29	**0.089**	**0.052**	0.25	0.15
Weakly stratified	**0**.**13**	**0**.**049**	−0.028	0.22	0.03	0.48	**0.13**	**0.076**	–0.046	0.33	0.0038	0.89
Weakly stratified#	**0**.**40**	**0**.**002**	−0.046	0.42	–0.015	0.7	**0.41**	**0.004**	–0.036	0.65	–0.026	0.96

*Var. represents the average explainable variance from three-way variation partitioning. P represents the p-value. Bold values indicate a significant (P < 0.05) or marginally significant (P < 0.1) result. # The analysis was done without the potential outlier (indicated in Figure 4).*

During weakly stratified periods, the environmental effect ([Env]) was the only component explaining vertical beta diversity (*adjusted R*^2^ = 13%, *P* = 0.049, Figure 4 and Table 1). Specifically, the environmental effect ([Env]) contributed by the pure effect of physicochemical dissimilarity ([Env|Dist+BF]) (Table 1). We noted, when one potential outlier was removed (Figure 4 and Table 1), physicochemical dissimilarity explained much more variance (*adjusted R*^2^ = 40%, *P* = 0.02, Table 1). These results suggested that during the weakly stratified periods, the effect of dispersal limitation diminished, and the vertical beta diversity was mainly determined by environmental effects.

## Discussion

### Mechanism Shaping Vertical Beta Diversity Varied Depending on Water Stratification

The mechanisms driving the vertical beta diversity of bacterioplankton in the Kuroshio east of Taiwan changed among seasons, depending on the strength of stratification. While the environmental effect was important in both strongly and weakly stratified periods (Figure 4), variation partitioning analysis indicated that the pure effect of physicochemical dissimilarity was significant only during weakly stratified periods (Table 1). These results suggested that factors other than geographic distance or stratification have caused the vertical physicochemical dissimilarity. An emergent question is, what caused this vertical physicochemical dissimilarity during the weakly stratified periods? Further investigation indicated that upwelling events were observed in the Kuroshio east of Taiwan during the weakly stratified periods (Supplementary Figure S1). Upwelling events have been recognized as an important to nutrient re-supply which brings up extra nutrients from the deeper layers to the euphotic zone (Capone and Hutchins, 2013). Indeed, we found an elevation of nutrients in the DCM compared to surface layers (Supplementary Figure S1), and the difference in nutrients (silicate and phosphate) concentration between surface and DCM were correlated with physicochemical dissimilarity (Supplementary Figure S2). That is, our results indicate that when stratification is weak, other physical factors such as upwelling can introduce a vertical physicochemical gradient, thus further influencing the vertical beta diversity. In contrast, during the strong stratification periods, the pure effect of physicochemical dissimilarity was not significant; rather, the physicochemical dissimilarity driving vertical beta diversity was mainly mediated by water stratification and vertical distance (multivariate linear regression, *adjusted R*^2^ = 45%, *P* = 0.029). In fact, it is well known that physicochemical dissimilarity increases with greater vertical distance (Legendre et al., 2005). Importantly, stratification strength played an additional role in affecting physicochemical dissimilarity, beyond the effect of vertical distance. This finding supports our hypothesis, and is consistent with general notions about how vertical beta diversity is determined under strong stratification. For instance, previous studies observed that strong water column stratification led to an enhanced gradient of physicochemical properties such as nutrients (Galand et al., 2010; Li et al., 2018). Thus, stratification in combination with vertical distance could enhance the physicochemical gradient, resulting in increased variation in bacterioplankton composition.

In addition, stratification strength also mediated the role of the spatial effect. In particular, the spatial effect was only observed when the stratification strength was strong (Table 1 and Supplementary Figure S4). These results support our hypothesis, indicating that the stratification did indeed create dispersal barriers for bacterioplankton, and thus influenced the vertical beta diversity through the spatial effect. Previous studies have mostly found that the spatial effect was significant at small (0–1 km) or at very large scales (>5000 km) (Hanson et al., 2012), suggesting that the spatial effect is scale dependent. In this study, we showed that even under at the same scale, the spatial effect can change through time, depending on the strength of stratification. These findings are in line with the notion that the geographic distance *per se* may not serve as a good proxy for dispersal limitation (Lindstrom and Langenheder, 2012); rather, hydrographic processes such as the connectivity between water masses would play a key role in mediating dispersal of bacteria (Galand et al., 2010; Hernando-Morales et al., 2017).

While we have thus far focused mainly on analyzing bacterioplankton at the community level, it will be illuminating to also examine at the population level for individual OTUs. Previous studies have showed that different OTU populations can respond differently to spatial and environmental effects (Langenheder and Lindstrom, 2019). Such differential responses may be ascribed to the difference in OTU abundances. For example, OTUs with lower abundances may have a lower chance to disperse; as such, spatial effects may have higher impacts on less abundant OTUs (Yang et al., 2016; Zhang et al., 2018). Moreover, it has been reported that OTUs with low abundance also have narrower niche breadths compared to their abundant counterparts (Wu et al., 2017). Hence, OTUs with lower abundances may show stronger responses to environmental effects. Indeed, in our study, we found that the spatial and environmental effects showed a larger impact on less abundant OTUs (Supplementary Figure S4). Such responses are especially evident, when those effects at the community level were significant (Table 1). That is, our study indicates that the mechanisms driving vertical beta diversity at the community level were mainly mediated by those OTUs with low abundance.

In our study, we found that the strength of stratification alters the mechanisms determining vertical beta diversity of bacterioplankton. These findings can be further integrated into the metacommunity theory. Metacommunity theory suggests that beta diversity is shaped by the interplay between environmental (i.e., local) and spatial (i.e., regional) factors (Leibold et al., 2004). Here, we found that physicochemical dissimilarity and/or dispersal barriers can be introduced by stratification strength. Our study provides quantitative evidence supporting the previous studies, suggesting that the relative importance of local versus regional processes of bacterial metacommunities changes depending on the degree of mixing/stratification among water masses (Galand et al., 2010; Yeh et al., 2015). Moreover, as previous studies have emphasized, the beta diversity of a community and its underlying assembly processes would impact ecosystem functioning (Leibold et al., 2017). In this context, potential ocean warming which can increase stratification warrants further investigation on its effect on vertical beta diversity of marine bacterioplankton and ecosystem functioning.

### The Comparison of Vertical Beta Diversity

Here we note, that although the mechanisms driving bacterioplankton vertical beta diversity differed between strong and weak stratification periods, there was no significant difference in mean vertical beta diversity of bacterioplankton assemblages between these two periods (Figure 3B). This result is consistent with a study that observed a large variation in vertical beta diversity during winter (i.e., weakly stratified periods) (Chow et al., 2013) but contradicts other studies which have indicated that the vertical gradient of beta diversity should be weakened during mixing periods (Treusch et al., 2009; Giovannoni and Vergin, 2012). These contradictory results can be attributed to the difference among local hydrographic conditions. For example, in the Kuroshio east of Taiwan, upwelling events were observed during the mixing periods. Since upwelling events can generate a large range of environmental gradients among sites (Figure 2), such appreciable environmental gradients can enhance vertical beta diversity, even during the mixing periods (Figure 4 and Supplementary Figure S2).

### Implications for Further Studies on Vertical Beta Diversity

This study highlights the importance of investigations into bacterioplankton beta diversity in the vertical dimension as well as identifying different mechanisms shaping vertical beta diversity depending on water column physics. However, a few caveats should be borne in mind. First, albeit sampling bacterial communities in surface and DCM layers may be a cost-effective way to capture variation for vertical beta diversity (Giovannoni and Vergin, 2012; Chow et al., 2013), this sampling strategy may generate confounding effects. For example, the effect of physicochemical dissimilarity might be confounded with the effect of vertical distance, since the vertical distance was determined by the depth of DCM that is highly sensitive to nutrient supply from the deeper layer (Estrada et al., 1993). Therefore, to have a better understanding of how beta diversity was determined by physicochemical dissimilarity, vertical distance, and strength of stratification, sampling from multiple depths should be considered. Second, while stratification strength can be a proxy of a barrier to dispersal, it cannot capture other physical processes affecting the water column (e.g., upwelling, downwelling, and eddies). Thus, future studies should also consider other physical processes. Finally, a large proportion of variation remains unexplained for vertical beta diversity in both strong and weak stratification periods. The unexplained variation may be attributed to unmeasured variables. For instance, environmental variables such as dissolved and particulate organic carbon have been implicated as important resources for many marine bacteria (Azam, 1998; Simon et al., 2002). Furthermore, biological variables such as viruses (Cram et al., 2015) and flagellates (Yang et al., 2018) are known to control bacterial community structure through prey-predator interactions. These variables need to be considered in future studies.

## Conclusion

Here we showed that the key mechanism determining vertical beta diversity varied among seasons in the Kuroshio region east of Taiwan, depending on strength of stratification. During weakly stratified periods, the spatial effect was diminished and the environmental effect became the only mechanism driving vertical beta diversity. In contrast, during strongly stratified periods, the vertical beta diversity was determined by both spatial and environmental effects. Importantly, stratification strength played an important role in affecting physicochemical dissimilarity and/or creating dispersal barriers. These findings highlight the importance of considering vertical hydrographical properties for studying metacommunity structure in marine ecosystems. More importantly, as climate changes such as warming can intensify water column stratification (Lyman et al., 2010), warming can potentially affect vertical beta diversity of microbes, which in turn likely affects ecosystem functioning in the ocean.

## Data Availability Statement

The datasets generated for this study can be found in the NCBI Sequence Read Archive (SRA) under the accession number: PRJNA543843 and Supplementary Data Sheet 1.

## Author Contributions

W-HC and CH conceived the research idea. W-HC, C-CC, and SJ collected the data. WC analyzed the data with assistance from H-PL and CH. W-HC, H-PL, and CH wrote the manuscript with the comments from co-authors.

## Conflict of Interest

The authors declare that the research was conducted in the absence of any commercial or financial relationships that could be construed as a potential conflict of interest.

## Footnotes

1 http://www.r-project.org/
