# Supplementary material for: Vertical Beta-Diversity of Bacterial Communities Depending on Water Stratification
Source: Front Microbiol. 2020 Mar 19;11:449. doi: 10.3389/fmicb.2020.00449 (PMC7103642; doi:10.3389/fmicb.2020.00449)
Supplement: Supplementary file 1 [file Table_1.docx]

TABLE S1| Values of the vertical beta diversity, physicochemical dissimilarity, vertical distance and the strength of stratification are listed for each paired surface and deep chlorophyll maximum (DCM) stations (c.f. Figure 1).

| Cruise | Station | Vertical beta diversity | Physicochemical dissimilarity | Vertical distance | Maximum Buoyancy frequency | Periods |
| --- | --- | --- | --- | --- | --- | --- |
| Jun_2013 | 2 | 0.54065763 | 4.32623412 | 36 | 0.00149271 | Strongly stratified |
| Jun_2013 | 3 | 0.50830339 | 3.15786988 | 21 | 0.00121912 | Strongly stratified |
| Jun_2013 | 4 | 0.61751017 | 6.08115303 | 26 | 0.00226696 | Strongly stratified |
| Jun_2013 | 6 | 0.29902881 | 4.32717853 | 66 | 0.0008016 | Strongly stratified |
| Mar_2014 | 1 | 0.74657627 | 4.4252969 | 31 | 1.30E-04 | Weakly stratified |
| Mar_2014 | 2 | 0.52045254 | 1.34976204 | 46 | 9.52E-06 | Weakly stratified |
| Mar_2014 | 4 | 0.51050848 | 1.63537396 | 26 | 4.11E-05 | Weakly stratified |
| Mar_2014 | 5 | 0.56524915 | 4.4443454 | 26 | 4.62E-05 | Weakly stratified |
| Mar_2014 | 7 | 0.43761695 | 0.96800331 | 66 | 1.55E-05 | Weakly stratified |
| Mar_2014 | 8 | 0.48501864 | 0.75563763 | 66 | 8.98E-05 | Weakly stratified |
| Jul_2014 | 1 | 0.40307627 | 3.75435286 | 26 | 0.00174741 | Strongly stratified |
| Jul_2014 | 2 | 0.34112034 | 1.22224939 | 10 | 0.00182098 | Strongly stratified |
| Jul_2014 | 3 | 0.38551356 | 4.84814137 | 46 | 0.00154725 | Strongly stratified |
| Jul_2014 | 4 | 0.39902712 | 3.49119775 | 76 | 0.00095186 | Strongly stratified |
| Jul_2014 | 5 | 0.29993729 | 2.64365762 | 46 | 0.00147229 | Strongly stratified |
| Jul_2014 | 6 | 0.80105763 | 5.09037509 | 171 | 0.00076754 | Strongly stratified |
| Jul_2014 | 7 | 0.28137627 | 2.54170125 | 71 | 0.00085227 | Strongly stratified |
| Jul_2014 | 8 | 0.47358475 | 2.46240465 | 106 | 0.00076892 | Strongly stratified |
| Nov_2014 | 1 | 0.35164746 | 1.73220072 | 33 | 0.00071476 | Weakly stratified |
| Nov_2014 | 2 | 0.31109322 | 0.13034744 | 12 | 0.00016361 | Weakly stratified |
| Nov_2014 | 3 | 0.52315932 | 0.80433872 | 25 | 0.0001697 | Weakly stratified |
| Nov_2014 | 4 | 0.58648814 | 1.08719178 | 55 | 0.00042527 | Weakly stratified |
| Nov_2014 | 5 | 0.29910848 | 1.07375294 | 72 | 0.0002139 | Weakly stratified |
| Nov_2014 | 6 | 0.47099153 | 1.34962254 | 95 | 0.0018661 | Weakly stratified |
| Nov_2014 | 7 | 0.52251356 | 1.80455715 | 103 | 0.00048625 | Weakly stratified |
| Nov_2014 | 8 | 0.53744407 | 2.43126771 | 102 | 0.00070114 | Weakly stratified |
| Mar_2015 | 1 | 0.81868644 | 0.22395319 | 26 | 5.93E-06 | Weakly stratified |
| Mar_2015 | 2 | 0.68600848 | 2.96907706 | 16 | 6.55E-06 | Weakly stratified |
| Mar_2015 | 3 | 0.42501695 | 1.87792677 | 56 | 4.60E-04 | Weakly stratified |
| Mar_2015 | 4 | 0.58326102 | 2.49017236 | 86 | 1.54E-04 | Weakly stratified |
| Mar_2015 | 5 | 0.54231186 | 2.18543316 | 86 | 1.69E-04 | Weakly stratified |
| Mar_2015 | 6 | 0.1694339 | 1.02517436 | 76 | 1.17E-04 | Weakly stratified |
| Mar_2015 | 7 | 0.39102712 | 0.48018892 | 61 | 1.28E-04 | Weakly stratified |
| Mar_2015 | 8 | 0.35193898 | 1.58744869 | 91 | 3.42E-04 | Weakly stratified |
