# Supplementary material for: Vertical Beta-Diversity of Bacterial Communities Depending on Water Stratification
Source: Front Microbiol. 2020 Mar 19;11:449. doi: 10.3389/fmicb.2020.00449 (PMC7103642; doi:10.3389/fmicb.2020.00449)
Supplement: Supplementary file 2 [file Data_Sheet_1.docx]

Supplementary Material

# Supplementary Figures and Table


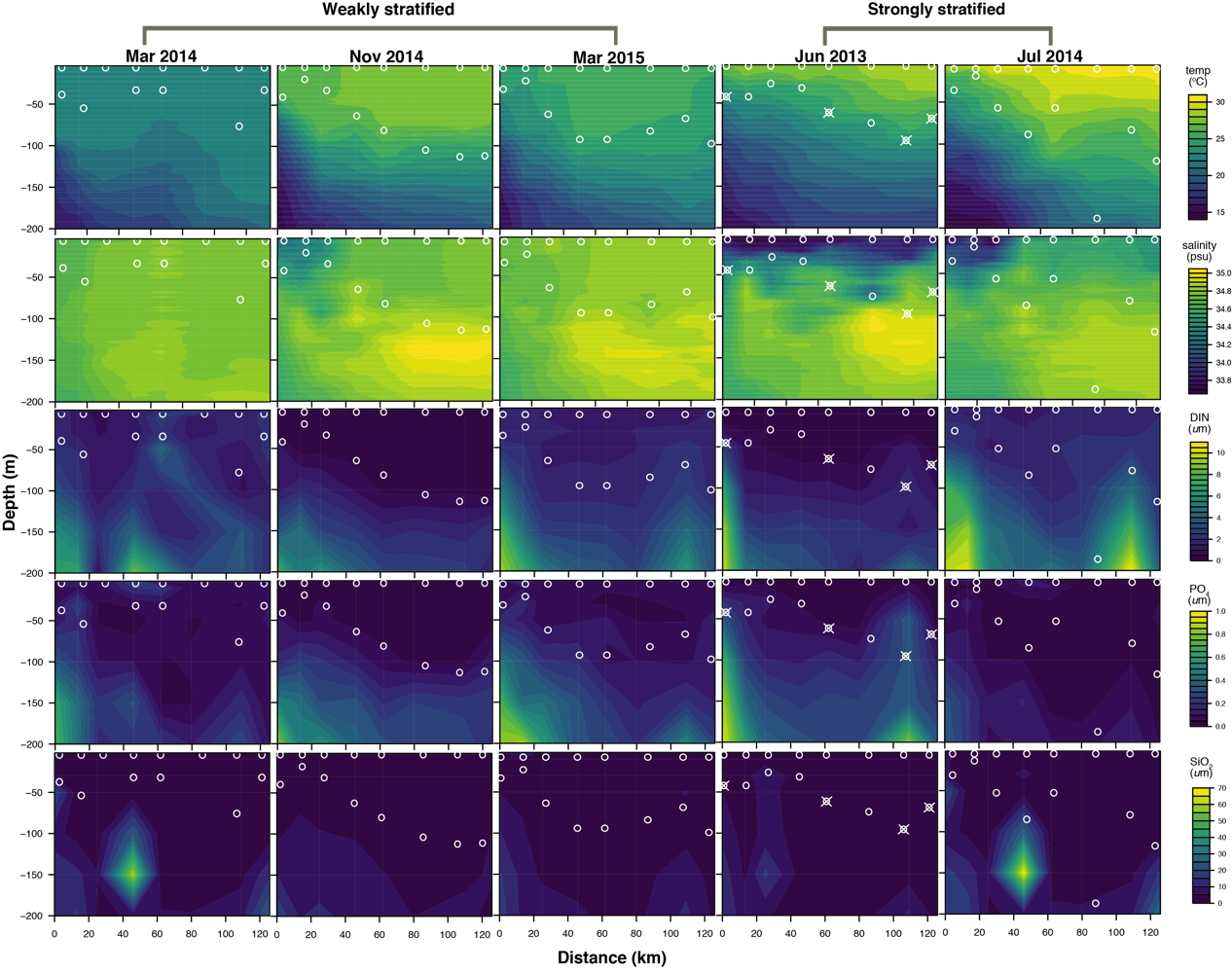


**Supplementary Figure 1.** Hydrographic properties along the sampling transect for different sampling periods for chlorophyll *a* concentration ($\mu$g/L), DIN ($\mu M$), SiO_2_ ($\mu M$), PO_4_ ($\mu M$), temperature ($℃)$ and salinity (psu) within 200 m water depth. Circles indicate sampling locations (surface and deep chlorophyll *a* maximum layers). Circles combined with crosses indicate samples with contamination (see Figure S3); therefore, these contaminated sampling events and their corresponding surface samples were both excluded from the rest of analysis.

**
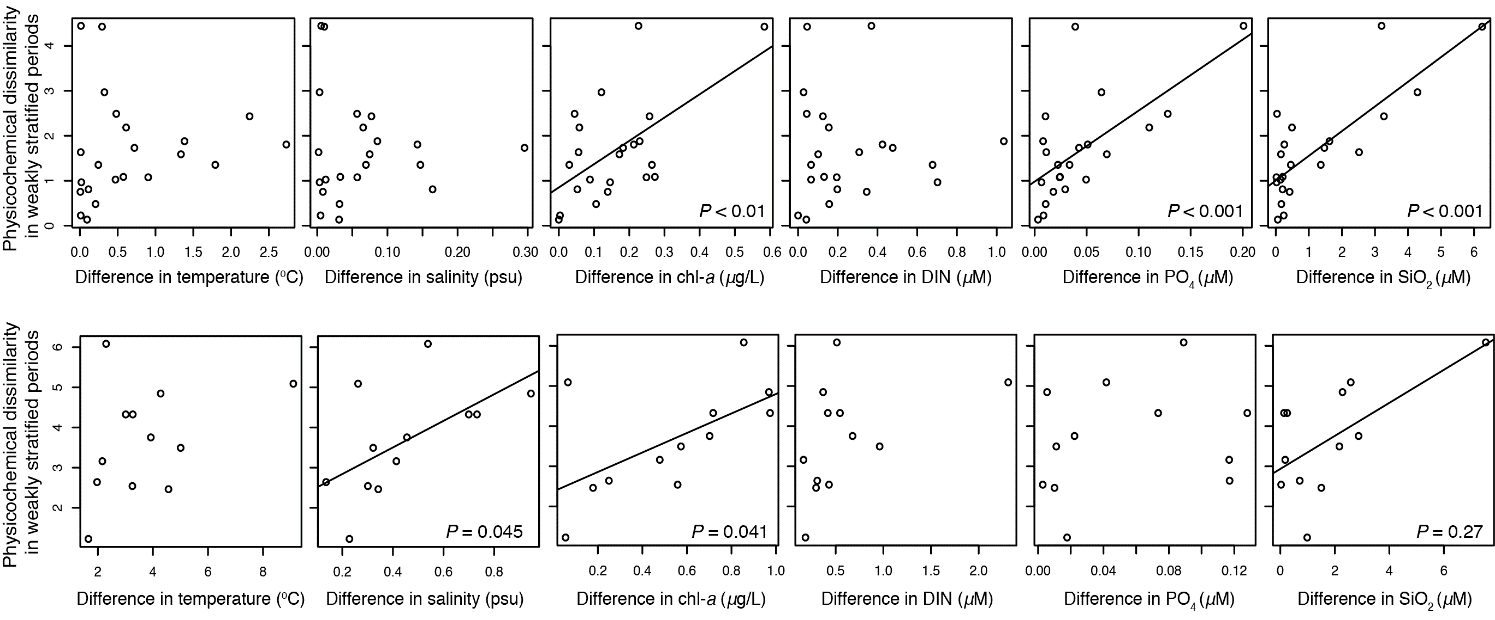
**

**Supplementary Figure 2.** Physicochemical dissimilarity of paired surface and deep chlorophyll maximum (DCM) communities in relation to the difference in temperature ($℃)$, salinity (psu), chlorophyll *a* concentration ($\mu$g/L), DIN ($\mu M$), PO_4_ ($\mu M$) and SiO_2_ ($\mu M$) of the corresponding paired surface and DCM sampling depths. The solid lines represent a significant relationship.


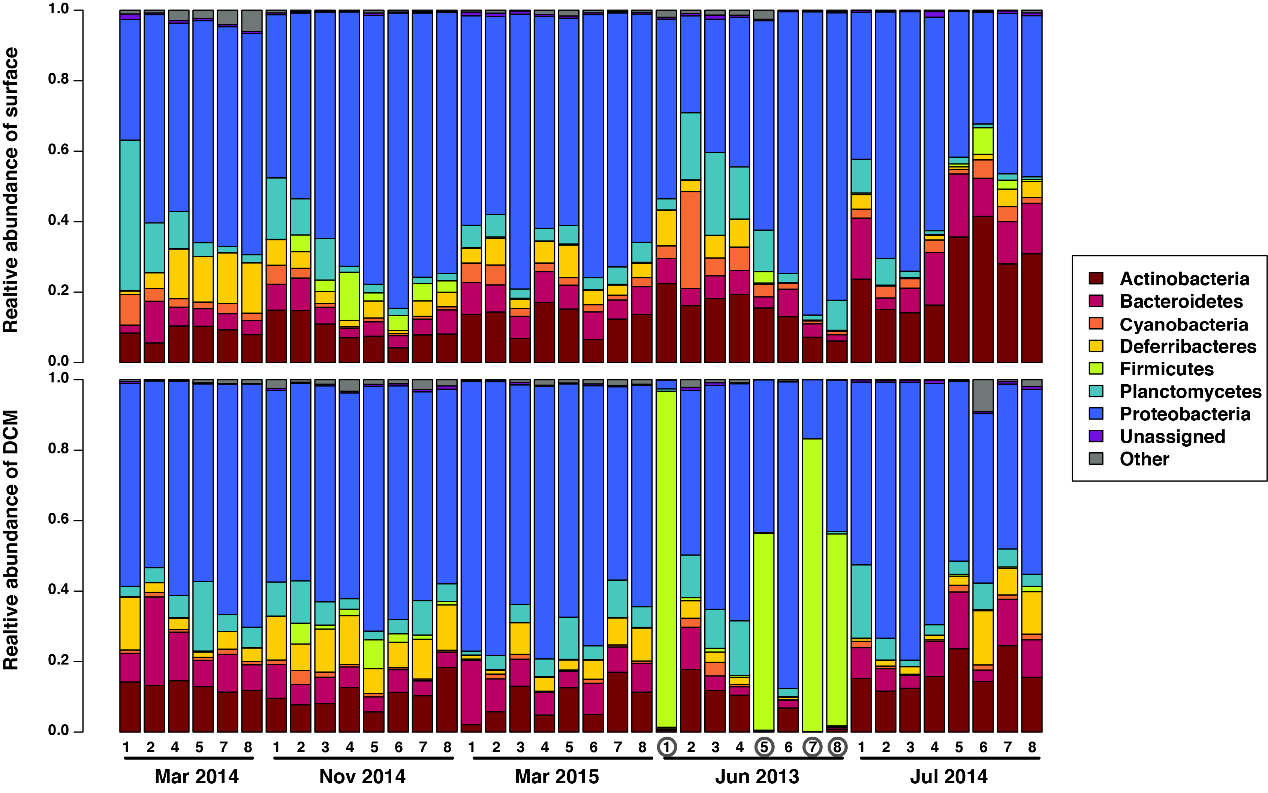


**Supplementary Figure 3.** Taxonomic composition for each sampling site along the transect of the Kuroshio region east of Taiwan. The circled sites were excluded from the rest of the analysis given the potential contamination according to taxonomic composition.


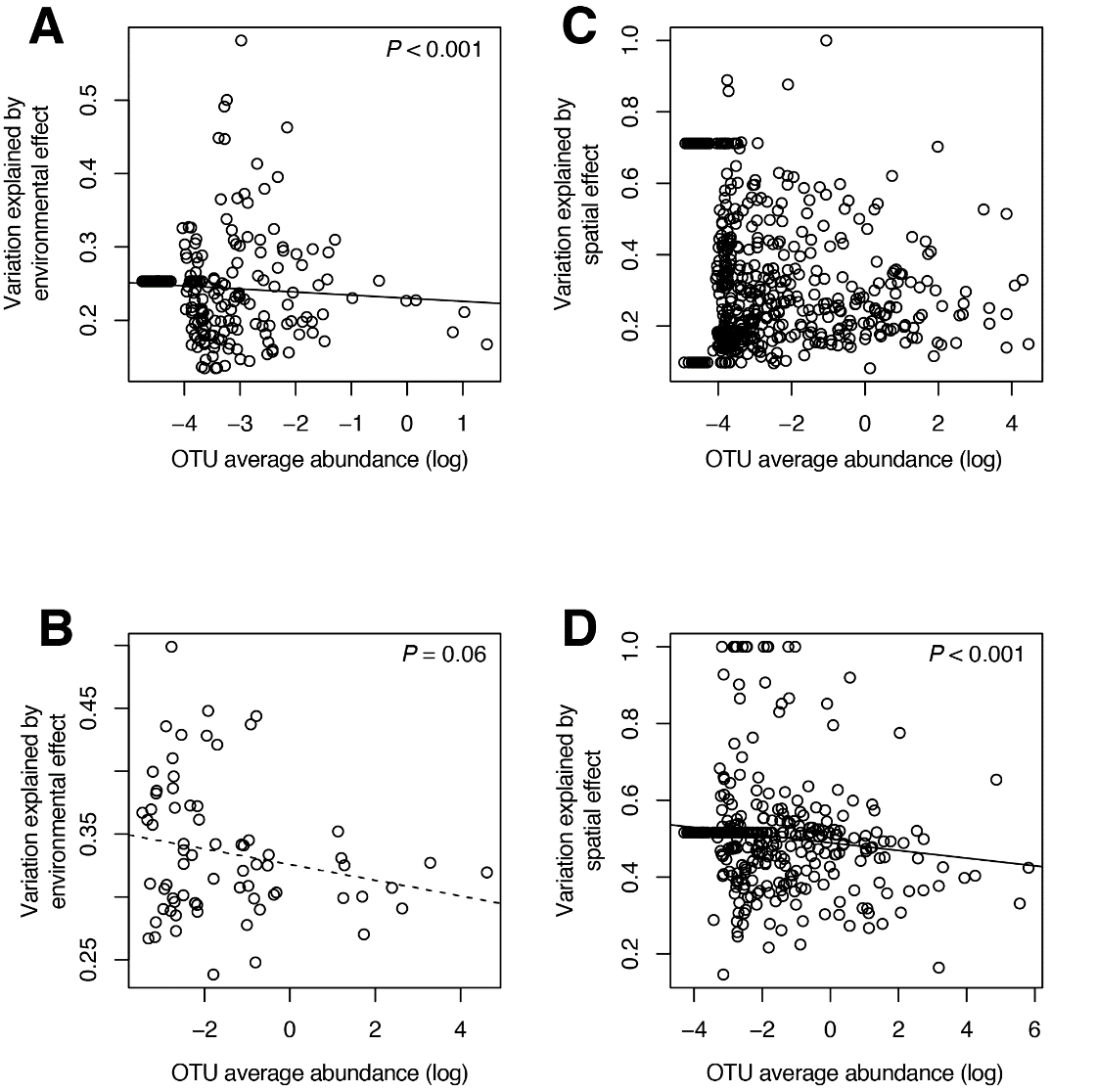


**Supplementary Figure 4.** Effects of OTU abundance on variation partitioning. The paired differences between the abundance of each OTU in the surface and DCM layers during the weakly (A and C) and strongly (B and D) stratified periods were analyzed with three-way partitioning. The three-way partitioning decomposed the variation in the abundance difference of each OTU into a pure physicochemical dissimilarity component ([Env$|$Dist$+$BF]), a pure stratification component ([BF$|$Env$+$Dist]), and a pure distance component ([Dist$|$Env$+$BF]). Here, the environmental effect (A and B) is represented by physicochemical dissimilarity ([Env]), while the spatial effect (C and D) is represented by pure stratification component ([BF$|$Env$+$Dist]) and pure distance component ([Dist$|$Env$+$BF]). Only the OTUs exhibiting significant results are selected and shown in each panel. Then, linear regression was employed to examine whether a relationship exists between the explained variation versus the OTU abundance. The solid and dashed line represent a significant relationship (*P* < 0.001) and marginally significant relationship (*P* < 0.1), respectively.
